# What is the minimum time interval for reporting of intraoperative core body temperature measurements in pediatric anesthesia? A secondary analysis

**DOI:** 10.1007/s10877-024-01254-y

**Published:** 2024-12-19

**Authors:** Clemens Miller, Anselm Bräuer, Johannes Wieditz, Marcus Nemeth

**Affiliations:** 1 Department of Anesthesiology, University Medical Center Goettingen, Robert-Koch-Straße 40, 37075 Goettingen, Germany; 2 Department of Anesthesiology, Children’s Orthopedic Hospital Aschau im Chiemgau, Bernauer Straße 18, 83229 Aschau im Chiemgau, Germany; 3 Department of Medical Statistics, University Medical Center Goettingen, Humboldtallee 32, 37073 Goettingen, Germany

**Keywords:** Anesthesia, Body temperature, Pediatric, Temperature monitoring, Warming management

## Abstract

Given that perioperative normothermia represents a quality parameter in pediatric anesthesia, numerous studies have been conducted on temperature measurement, albeit with heterogeneous measurement intervals, ranging from 30 s to fifteen minutes. We aimed to determine the minimum time interval for reporting of intraoperative core body temperature across commonly used measurement intervals in children. Data were extracted from the records of 65 children who had participated in another clinical study and analyzed using a quasibinomial mixed linear model. Documented artifacts, like probe dislocations or at the end of anesthesia, were removed. Primary outcome was the respective probability of failing to detect a temperature change of 0.2 °C or more at any one measurement point at 30 s, one minute, two minutes, five minutes, ten minutes, and fifteen minutes, considering an expected probability of less than 5% to be acceptable. Secondary outcomes included the probabilities of failing to detect hypothermia (< 36.0 °C) and hyperthermia (> 38.0 °C). Following the removal of 4,909 exclusions, the remaining 222,366 timestamped measurements (representing just over 60 h of monitoring) were analyzed. The median measurement time was 45 min. The expected probabilities of failing to detect a temperature change of 0.2 °C or more were 0.2% [95%-CI 0.0-0.7], 0.5% [95%-CI 0.0-1.2], 1.5% [95%-CI 0.2–2.6], 4.8% [95%-CI 2.7–6.9], 22.4% [95%-CI 18.3–26.4], and 31.9% [95%-CI 27.3–36.4], respectively. Probabilities for the detection of hyperthermia (*n* = 9) were lower and omitted for hypothermia due to low prevalence (*n* = 1). In conclusion, the core body temperature should be reported at intervals of no more than five minutes to ensure the detection of any temperature change in normothermic ranges. Further studies should focus on hypothermic and hyperthermic ranges.

## Clinical trial registration

This study is a secondary analysis from a prospective cohort study, which was registered in the German Clinical Trials Register (DRKS00024703).

## Introduction

Maintaining perioperative normothermia is one of the 10 N quality metrics in children [1], making it a key component of pediatric anesthesia care. This requires an appropriate warming strategy and an accurate measurement of body core temperature [2]. While the former can be efficiently achieved by modern warming systems such as forced-air warming [3, 4], the latter remains a subject of discussion because the ideal thermometry for children has not yet been identified [2, 5].

Therefore, several studies have been conducted to evaluate the efficacy of non-invasive thermometers compared to common and established measurement reference sites, such as the esophagus [5–9] or the rectum [9, 10]. Others have evaluated the accuracy of measurements at the nasopharyngeal site [11–13]. Although the results are somewhat inconsistent and reveal specific weaknesses that need to be considered, it is apparent that there were discrepancies in the measurement approaches, particularly with regard to the intervals used for temperature measurements. While most studies reported a one-minute interval [5, 7, 11], others used a thirty-second [8], two-minute [9, 10], five-minute [6, 12], or even fifteen-minute interval [13].

Continuous measurement of core body temperature is considered a standard of care in pediatric anesthesia [14]. However, there is a paucity of evidence regarding the minimum interval required for the reporting of intraoperative core temperature. With the hypothesis that a shorter measurement interval does not equate to better accuracy, the objective of this study was to determine the probabilities of failing to detect intraoperative changes in core body temperature across different temperature measurement time intervals in children undergoing general anesthesia. Therefore, a probability of less than 5% was deemed acceptable. This was achieved through a secondary analysis of a prospective clinical study.

## Methods

This secondary analysis included individual patient data from a prospective, single-center observational study, evaluating the agreement of the non-invasive Temple Touch Pro Temperature Monitoring System skin sensor against standard esophageal core temperature in children [5]. The study was approved by the Institutional Review Board of the University Medical Center Goettingen, Goettingen, Germany (No. 22/2/21 on February 25th 2021) and registered in the German Clinical Trials Register (ID: DRKS00024703 on March 4th 2021). The recruitment period was from April 13th to July 15th 2021. Written informed consent from the legal guardians as well as children’s assent were obtained prior to enrollment. We adhered to the STROBE guidelines for reporting of observational studies [15].

### Subject selection and protocol

The initial study included data from 100 children (32 girls) aged 6 years or younger who underwent surgery under general anesthesia with a mean core body temperature measurement time of 68 min. The clinical procedure followed our standard of care warming strategy, including prewarming the operating room, keeping the children dressed until induction of anesthesia, and placing them on a warming blanket continuously heated by forced air. As a reference method for core body temperature, an esophageal probe (RÜSCH Pharyngeal Temperature Sensor™, Teleflex Medical, Athlone, Ireland) was placed after securing the airway. It was inserted according to the Whitby and Dunkin formula, aiming to place the tip of the probe in the distal fourth of the esophagus [16]. To transfer temperature values into a database, we used the VSCapture tool (version 1.002) [17, 18] and recorded pairs of temperature until both devices were removed before emergence from anesthesia. For the majority of patients (*n* = 65), temperature values were recorded at one-second intervals. However, for a subset of patients, values were only recorded at one-minute intervals due to data transmission errors. Detailed information on inclusion and exclusion criteria, the study protocol and the patient characteristics can be found in the study results publication [5].

### Data processing and outcomes

To best determine the probabilities of failing to detect temperature changes across different measurement time intervals, data were extracted only from the 65 patients whose core body temperature was recorded at one-second intervals. Artifacts in the electronic data always correlated with cases of probe dislocation or with the end of anesthesia and probe removal. These events were documented in the study protocol and manually corrected during data preparation. A missed change in core body temperature was defined as a deviation of 0.2 °C or more at any one measurement point when compared to the previous measurement point. This was derived for pragmatic reasons, as changes of 0.1 °C can occur at any time but should be recognized by providers before the next change of 0.1 °C occurs. The time intervals analyzed were selected based on their common usage or pragmatic relevance in daily routines. These included 30 s, one minute, two minutes, five minutes, ten minutes, and fifteen minutes. For each interval analyzed, the first measurement was designated as temperature value for the entire interval, as this approach is analogous to the actual measurement of temperature at the corresponding time intervals.

The **primary outcome** was the respective probability of failing to detect a temperature change for each time interval under analysis, considering an expected probability of less than 5% to be acceptable. **Secondary outcomes** were the probabilities of failing to detect hypothermia and hyperthermia. According to perioperative temperature management guidelines, hypothermia was defined as a core body temperature below 36.0 °C and hyperthermia as above 38.0 °C [19, 20]. Such an event was considered to have occurred if a patient was normothermic at the beginning of an interval but became hypothermic or hyperthermic during the course of the interval.

### Statistical analysis

As this study was a secondary analysis, no separate and specific sample size calculation was performed. Descriptive statistics were reported as numbers or median [1st − 3rd quartile] as appropriate. If not stated otherwise, tests were performed two-sided at a 5% significance level. Parameter estimates are provided with corresponding 95% confidence intervals [95%-CI].

For statistical analysis, we divided the measurement series for different measurement intervals into separate intervals of corresponding lengths and computed the number of events for each patient. We fitted a quasibinomial mixed linear model for the event probabilities with fixed effects measurement frequency and random individual effect. We used an identity link as link function to determine absolute risk reductions (ARR) as effect measures. 95% confidence intervals are based on a profile likelihood approximation. Test results were adjusted for multiple comparisons using Sidak’s method if applicable. Data were analyzed using R version 4.3.3 (The R Foundation for Statistical Computing, Vienna, Austria).

## Results

**Fig. 1:**
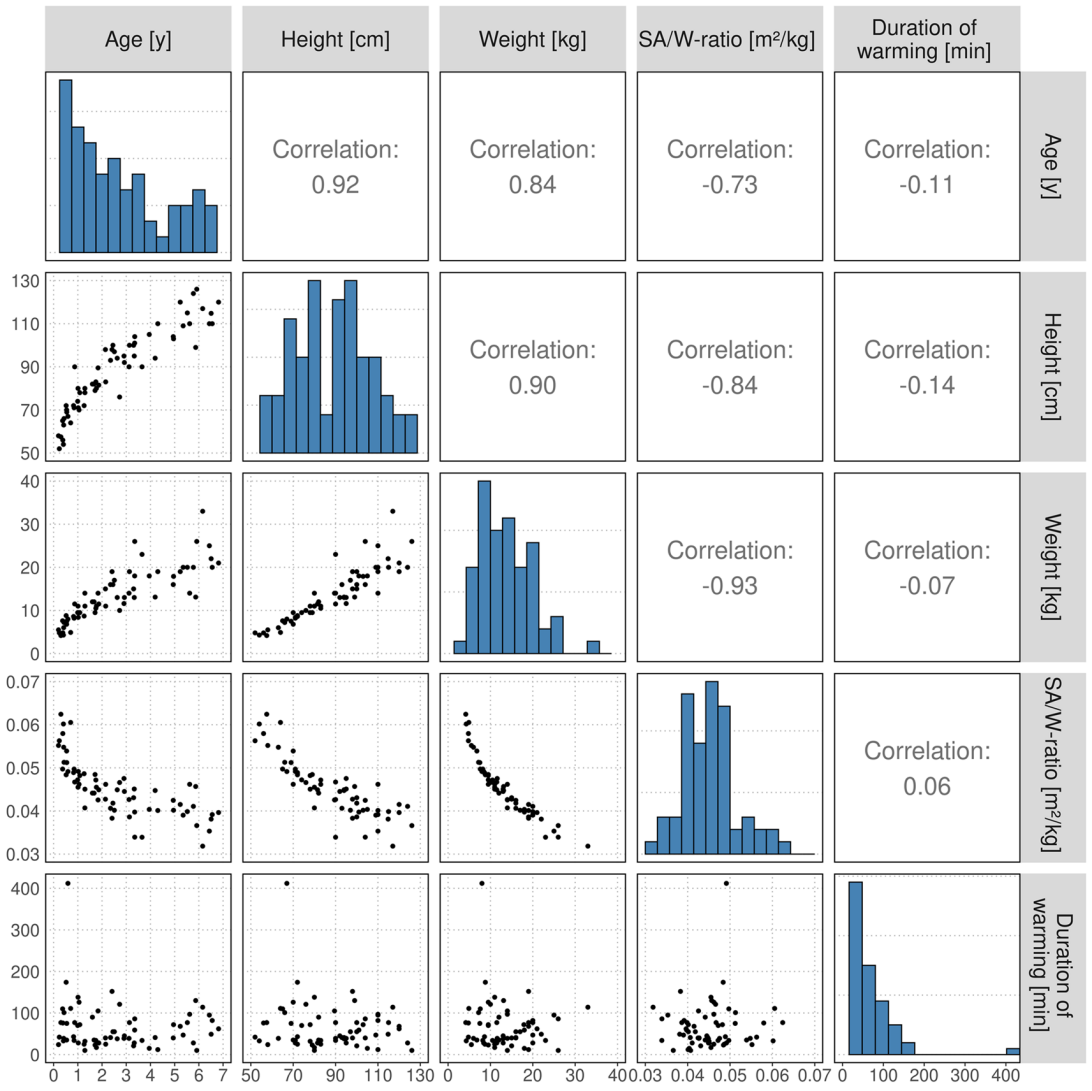
Scatterplot matrix of patient demographic parameters and duration of warming (lower left) with corresponding distributions (diagonal) and pairwise Pearson correlation (upper right)

Out of 227,275 timestamped measurements at a per-second level, 502 were excluded because of obvious probe removal at the end of anesthesia and 3,846 due to documented cases of probe dislocation. Further 561 timestamps were excluded because there were two or more measurements for the same timestamp. This resulted in a total of 222,366 measurements of 65 children (15 female) ranging in age from two months to 6.8 years being included in the final analysis. The actual duration of measurement was from 5 to 414 min. Further patient and data characteristics are presented in Table 1. Distributions and correlations between baseline patient characteristics are shown in Fig. 1. The numbers of observed episodes per interval and event are shown in Table 2.

**Table 1:** Patient and data characteristics. Values are numbers (%) or median [1st– 3rd quartile]

Patients	65 (15 female)
Age [y]	2.2 [1–4.0]
Height [cm]	88.1 [72–101]
Weight [kg]	13.5 [8.8–18]
Surface-area-to-weight ratio [m²/kg]	0.0452 [0.0404–0.0485]
Duration of warming [min]	45 [33–81]
Operating room temperature [°C] at start of measurement	25.3 [24.2–26.1]
Operating room temperature [°C] at end of measurement	24.6 [23.3–25.7]
Type of general anesthesia Totally intravenous Balanced	27 (42%) 38 (58%)
Type of surgery General or Urology Trauma or Orthopedic Neurosurgery	52 (80%) 7 (11%) 6 (9%)

**Table 2:** Number of observed episodes with deviations ≥ 0.2 °C, hyperthermia and hypothermia, respectively

No. of observed episodes per event type	Total no. of episodes	Deviation ≥ 0.2 °C	Hyperthermia	Hypothermia
Interval				
30 s	8,226	14	1,038	1
1 min	4,143	15	526	1
2 min	2,093	24	267	1
5 min	863	30	114	1
10 min	450	86	62	1
15 min	312	100	45	1

The probability of failing to detect a temperature change of 0.2 °C or more increased with the duration of the time interval (Table 3). For the 30-second interval, the expected probability was 0.2% [95%-CI 0.0-0.7]. For the one-minute interval, the expected probability was 0.5% [95%-CI 0.0-1.2], while it was 1.5% [95%-CI 0.2–2.6] for the two-minute interval, 4.8% [95%-CI 2.7–6.9] for the five-minute interval, 22.4% [95%-CI 18.3–26.4] for the ten-minute interval, and 31.9% [95%-CI 27.3–36.4] for the fifteen-minute interval, respectively. The absolute risk reductions from one measurement interval to the next are shown in Table 4.

### Detection of hypothermia and hyperthermia

Because the prevalence of intraoperative hypothermia was 1.5% (*n* = 1) and this period lasted less than thirty seconds, event probabilities and absolute risk reductions were not calculated.

The prevalence of intraoperative hyperthermia was 13.8% (*n* = 9). The estimated probability of failing to detect hyperthermia in these patients was 0.1% for 30-second interval, 0.2% for one-minute interval, 0.3% for two-minute interval, 0.8% for five-minute interval, 1.6% for ten-minute interval, and 2.3% for fifteen-minute interval, respectively. Profile likelihood-based 95%-confidence intervals are given in Table 3. The absolute risk reductions from one measurement interval to the next are shown in Table 4. The absolute risk reductions of the investigated intervals did not reach a level of statistical significance.

**Fig. 2:**
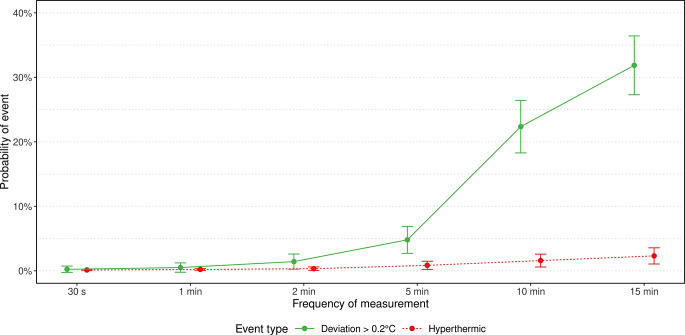
Mean probabilities (dots) of event with corresponding 95% confidence intervals (error bars). Colors indicate different event types: deviation ≥ 0.2 °C (green), hyperthermia (red)

Figure 2 depicts the event probability as a function of the selected reporting interval.

**Table 3:** Mean expected probabilities of failing to detect temperature changes for different measurement intervals with corresponding 95%-confidence intervals (in %)

Event type	Deviation ≥ 0.2 °C		Hyperthermia	
Interval	Expected probability [%]	95%-CI	Expected probability [%]	95%-CI
30 s	0.2	[0.0-0.7]	0.1	[0.0-0.2]
1 min	0.5	[0.0-1.2]	0.2	[0.0-0.4]
2 min	1.5	[0.2–2.6]	0.3	[0.1–0.6]
5 min	4.8	[2.7–6.9]	0.8	[0.2–1.5]
10 min	22.4	[18.3–26.4]	1.6	[0.6–2.6]
15 min	31.9	[27.3–36.4]	2.3	[1.1–3.6]

**Table 4:** Comparisons of mean event probabilities for consecutive measurement intervals (in %) stratified by event type. Reported is the absolute risk reduction (ARR), i.e. the reduction in the probability of experiencing an event, with corresponding 95%-confidence intervals (95%-CI) and p-values for testing for differences in mean event probabilities between measurement intervals

Event type	Deviation ≥ 0.2 °C			Hyperthermia		
Comparison	ARR [%]	95%-CI	p-value	ARR [%]	95%-CI	p-value
**1 min − 30 s**	0.3	[− 0.8–1.4]	0.978	0.1	[− 0.1–0.3]	0.669
**2 min–1 min**	0.9	[− 0.9–2.7]	0.634	0.1	[− 0.3–0.5]	0.925
**5 min–2 min**	3.4	[0.3–6.5]	0.025	0.5	[− 0.4–1.4]	0.497
**10 min–5 min**	17.6	[11.6–23.5]	< 0.001	0.7	[− 0.8–2.3]	0.694
**15 min–10 min**	9.5	[1.6–17.4]	0.010	0.7	[− 1.4–2.8]	0.897

## Discussion

The probability of failing to detect a change in children’s core body temperature intraoperatively increased with the length of the selected measurement interval. This primarily pertains to general alterations exceeding 0.2 °C, though it is of particular concern near the thresholds of hypothermia or hyperthermia.

The expected probability of failing to detect temperature changes of 0.2 °C or more was slightly below 5% when reporting a five-minute measurement interval. However, this result must be interpreted with caution, as it depends on the measurement intervals considered for this analysis. It is more likely to detect deviations of 0.2 °C or more for larger measurement intervals. Testing other measurement intervals between two and five minutes will likely shift the significance threshold for the absolute risk reductions in another direction. Regarding hyperthermic temperature ranges, we can see lower probabilities for the detection of hyperthermia than for detecting general deviations of 0.2 °C or more across different measurement intervals. We attribute this to the low duration and intensity of hyperthermia (13.8% with *n* = 9) in our patients, which may even underestimate the need for a small reporting interval in this subgroup. It seems reasonable that this also applies to the detection of hypothermia, although a definitive statement on this cannot be made due to the lack of sufficient data points in our cohort. Further analysis with a larger data set should be made on temperature deviations in the hypothermic range for a more valid statement. In order to address the optimal time interval for measuring and documenting core body temperature, our data do not allow any conclusions to be drawn that can be proven from statistical results alone. To derive practice-relevant recommendations, it is therefore necessary to integrate them with clinically plausible requirements. Nevertheless, the aim of this study was to examine measurement intervals, which were commonly reported in clinical studies in children [5–13]. A measurement interval of 3–4 min or anything in between was not used in any of these studies in children. In addition, defining a purely statistically determined interval such as 3.7 min is very unlikely to be part of in daily routine.

The maintenance of perioperative normothermia in pediatric patients requires an active warming management [2]. However, the currently available guidance on this topic is often insufficient. It is a fallacy to assume that establishing warming device settings and thereby achieving normothermia is a one-time process. Rather, it necessitates continuous monitoring and timely adjustments to the settings, particularly in instances where the core body temperature has not yet reached a stable equilibrium, which typically occurs only over an extended period [2]. This is corroborated by the evidence that there is a latency in the change in core body temperature following adjustments to the warming device [21]. It is therefore recommended that more stringent monitor alarm settings should be set for core temperature to account for this latency [21]. As a consequence, anesthesia providers should be alert for any changes in core body temperature, rather than merely react when the child’s temperature drops below 36 °C or rises above 38 °C. In view of this, we propose that a change of 0.2 °C can be considered relevant, especially if the temperature course is towards hypo- or hyperthermia. This is especially true for the surgical procedures with a high risk of iatrogenic hyperthermia in children [21], which now can occur even more common than hypothermia, as reported in other recent studies [4, 5, 22] and assumed by an expert group of pediatric anesthesiologists [23].

One might posit that the reporting interval should be as short as possible to avoid missing a temperature change. This is particularly important for scientific purposes. For these purposes, it would be ideal to measure core temperatures at intervals of no more than five minutes. This minimizes the probability of missing a relevant change in core temperature without collecting too many irrelevant data points. If digital documentation systems are used, this time interval could also be appropriate for generating automated quality control reports. However, the implementation of digital documentation systems is not yet universal [24]. In the case of analog and handwritten documentation, the situation is more complex. A temperature documentation interval of fifteen minutes is often used, given the limited space on a protocol as well as the limited time for documentation. However, one must be aware that this will have a probability of roughly 30% of missing a core temperature change of 0.2 °C. Thereby, extending the time interval to fifteen minutes introduces the potential for documentation gaps.

### Limitations

This study is subject to a number of limitations. First, the prevalence of intraoperative hypothermia in our cohort was too low for calculating data, so we omitted its analysis. This has a major impact on the interpretation of our data set, as the detection of hypothermia is an essential factor in perioperative temperature management. Second, the statistical threshold set for the expected probability of 5% was selected arbitrarily and therefore represents only a plausible order of magnitude. Third, we did not include the one-second measurement interval into the model for numerical reasons. This was because the events of interest almost never occurred at this measurement interval. As a result, comparisons with this interval would yield numerical instabilities and clinically hardly interpretable results. However, it is also unlikely that temperature changes of 0.2 °C or more occur within one second and are not attributable to dislocation. Fourth, a specific sample size calculation was not performed, as the results are derived and extrapolated from another study subject. Fifth, gender-specific measurement issues were not evaluated. Sixth, we did not account for issues with decimals in measurement software, although we do not believe that there are clinically relevant differences in rounding decimals of temperature measurements among different software.

## Conclusion

In children undergoing general anesthesia, the core body temperature should ideally be reported at intervals as short as possible to avoid missing a temperature change. For scientific purposes, the interval should be no more than five minutes to minimize the probability of missing a relevant change. This may also apply to digital documentation systems, the implementation of which would be advantageous. But in the case of analog and handwritten documentation, a balance must be struck due to the limited space on the protocol and the amount of work required. One must be aware that a fifteen-minute interval has a high probability of missing a change in core body temperature, where it may not be clear whether the warming settings should perhaps have been adjusted earlier. Further studies should focus on detection of changes in hypothermic and hyperthermic ranges.

## Data Availability

No datasets were generated or analysed during the current study.
